# Methyl Sartortuoate Inhibits Colon Cancer Cell Growth by Inducing Apoptosis and G2/M-Phase Arrest

**DOI:** 10.3390/ijms160819401

**Published:** 2015-08-17

**Authors:** Qiusheng Lan, Shoufeng Li, Wei Lai, Heyang Xu, Yang Zhang, Yujie Zeng, Wenjian Lan, Zhonghua Chu

**Affiliations:** 1 Guangdong Provincial Key Laboratory of Malignant Tumor Epigenetics and Gene Regulation, Department of Gastrointestinal Surgery, Sun Yat-sen Memorial Hospital, Sun Yat-sen University, Guangzhou 510120, China; E-Mails: lansysu@foxmail.com (Q.L.); weilaiv2001@126.com (W.L.); xuheyang2012@126.com (H.X.); zyykq1202@foxmail.com (Y.Zh.); sumsyorky@hotmail.com (Y.Ze.); 2 Department of Colorectal Surgery, Fujian Medical University Union Hospital, Fuzhou 350000, China; E-Mail: jesmy@126.com; 3 School of Pharmaceutical Sciences, Sun Yat-sen University, Guangzhou 510006, China

**Keywords:** methyl sartortuoate, colon cancer, apoptosis, G2-M arrest, MAPK

## Abstract

The potential anti-neoplastic activity of terpenoids is of continued interest. In this study, we investigate whether methyl sartortuoate, a terpenoid isolated from soft coral, induced cell cycle arrest and apoptosis in a human colon cancer cell line. Culture studies found that methyl sartortuoate inhibited colon cancer cell (LoVo and RKO) growth and caused apoptotic death in a concentration- and time-dependent manner, by activation of caspase-8, caspase-9, caspase-3, p53 and Bax, and inactivation of B-cell lymphoma 2 (Bcl-2) apoptosis regulating proteins. Methyl sartortuoate treatment led to reduced expression of cdc2 and up-regulated p21 and p53, suggesting that Methyl sartortuoate induced G2-M arrest through modulation of p53/p21/cdc2 pathways. Methyl sartortuoate also up-regulated phospho-JNK and phospho-p38 expression levels. This resulted in cell cycle arrest at the G2-M phase and apoptosis in LoVo and RKO cells. Treatment with the JNK inhibitor SP600125 and the p38 MAPK inhibitor SB203580 prevented methyl sartortuoate-induced apoptosis in LoVo cells. Moreover, methyl sartortuoate also prevented neoplasm growth in NOD-SCID nude mice inoculated with LoVo cells. Taken together, these findings suggest that methyl sartortuoate is capable of leading to activation of caspase-8, -9, -3, increasing p53 and Bax/Bcl-2 ratio apoptosis through MAPK-dependent apoptosis and results in G2-M phase arrest in LoVo and RKO cells. Thus, methyl sartortuoate may be a promising anticancer candidate.

## 1. Introduction

Colon cancer remains one of the primary world health concerns. It is one of the leading causes of mortality due to the absence of effective treatments and its unclear pathogenesis [1]. Apart from surgery, fluorouracil-based chemotherapy is widely used to treat advanced colon cancer but is associated with high toxicity and harmful side effects [2,3]. The discovery of novel preventative and effective therapeutic agents is an ongoing need to combat this disease. Marine natural products have been shown to have outstanding potential in the prevention and treatment of cancers, especially those of the gastrointestinal tract [4].

Terpenoids are secondary metabolites that occur in most living organisms and which have potential anticancer activity [5]. Terpenoids have been shown to prevent carcinogenesis and to act as intermediate biomarkers and indicators of premalignancy [6]. The terpenoid methyl sartortuoate is isolated from the soft coral Sarcophyton tortuosum Tix.-Dur. (Alcyoniidae) collected from Sanya Bay, Hainan Island in China [7].

Cells play an active role in their own biological death, which is known as apoptosis. The intrinsic pathway of apoptosis is regulated by the B-cell lymphoma 2 (Bcl-2) family proteins. A balance between pro-apoptotic (Bax, BID, BAK, or BAD) and anti-apoptotic (Bcl-XL, Bcl-2, BCLWor MCL1) proteins of the Bcl-2 family controls the mitochondrial apoptosis pathway [8]. Caspase-dependent apoptosis occurs through either an extrinsic pathway leading to activation of caspase-8 or via an intrinsic pathway leading to activation of caspase-9. Both pathways can form a crosstalk through caspase-8-mediated cleavage of Bid, resulting in its translocation to mitochondria and cytochrome c release to stimulate the intrinsic pathway [9]. All of them converge to a final common pathway involving the activation of effector caspases, like procaspase-3 in some cell lines, and finally lead to apoptosis.

Cell cycle control is one of the major regulatory mechanisms of cell growth. Many anticancer agents have been reported to arrest the cell cycle at a specific checkpoint and thereby induce apoptotic cell death [10]. The eukaryotic cell division cycle, to a great extent, is regulated by cyclin/cyclin-dependent kinase (CDK) complexes, which are in turn modulated by CDK inhibitors (CKIs), such as p21WAF1/Cip1 (referred to as p21 hereafter), that bind to specific cyclin/CDK complexes [11].

Mitogen activated protein kinase (MAPK) is an important intracellular signal transduction system and participates in a series of physiological and pathological processes, including cell growth, differentiation and apoptosis [12]. The most prominent members of the MAPKs family that correlated to apoptosis are c-Jun-N-terminal kinase (JNK) and p38 MAPK. Abundant evidence indicates that antitumor agents can alter the biological behaviors of MAPK members in most cancer cell lines [13].

There is evidence that cellular tumor antigen (p53) as one of the tumor suppressors has the ability to block the cell cycle, to initiate DNA repair, to accelerate cell ageing and to induce apoptosis [14]. Some anticancer studies proved that p53/p21/cdc2 pathways are modulated by some agents to induce G2/M arrest. Also p53 can induce apoptosis by up-regulation of Bax/Bcl-2 ratios [15]. The point is that when JNK and p38 pathways are activated, they can up-regulate p53 expression, leading to cell cycle arrest and apoptosis.

In this study, we investigated the antineoplastic activity of methyl sartortuoate against human advanced colon carcinoma cells and explored the possible mechanisms involved.

## 2. Results

### 2.1. Inhibition of Colon Cancer Cell Proliferation by Methyl Sartortuoate

**Figure 1:**
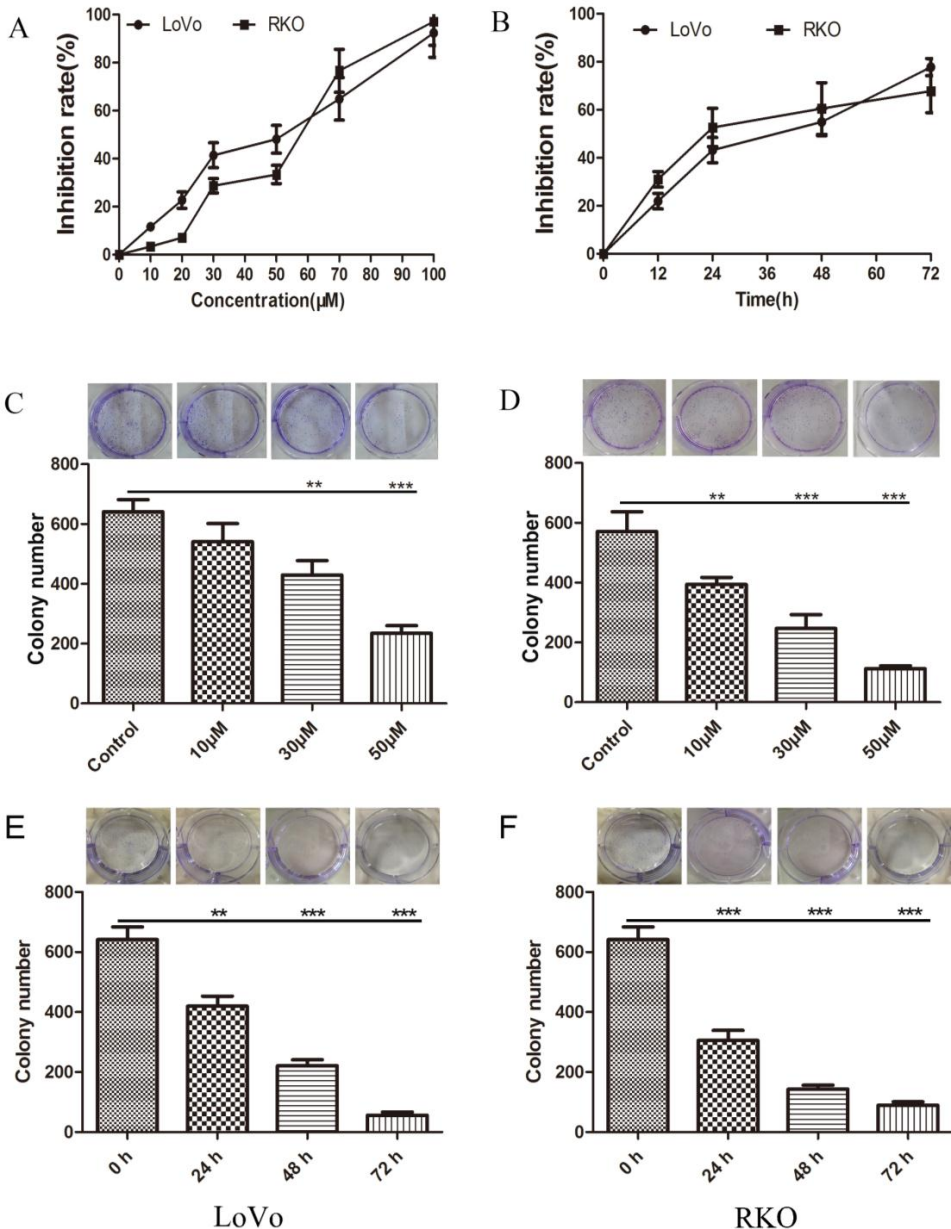
Methyl Sartortuoate induced cell proliferation inhibition in coloron cancer LoVo and RKO cells. (**A**) Cell inhibition rate was measured with MTT when LoVo and RKO cells were treated with Methyl Sartortuoate at indicated concentrations for 24 h; (**B**) LoVo and RKO cells were treated with Methyl Sartortuoate at 50 μM for different time intervals; (**C**,**D**) LoVo and RKO cell colony formation with or without Methyl Sartortuoate treatment. Results are presented as the average of quadruplicate measurements, and the bar is the standard deviation (*n* = 3). ** *p* < 0.01 and *** *p* < 0.001 *versus* control group; (**E**,**F**) LoVo and RKO cells colony formation with Methyl Sartortuoate treatment at 30 µM for 24, 48 and 72 h. Results are presented as the average of quadruplicate measurements, and the bar is the standard deviation (*n* = 3). ** *p* < 0.01 and *** *p* < 0.001 *versus* 0 h group.

Following 24 h exposure to methyl sartortuoate there was evidence of concentration-dependent cytotoxicity in LoVo and RKO cells (Figure 1A). LoVo and RKO cells exposed to 50 µM of methyl sartortuoate (*i.e.*, close to the estimated IC_50_ of 48 µM) for different periods of time suggested that the effect of methyl sartortuoate on cell growth was also time-dependent (Figure 1B). In addition, colony formation assays demonstrated that LoVo and RKO cells treated with different concentrations of methyl sartortuoate for 24 h formed both fewer and smaller colonies than did control colon cancer cells (Figure 1C,D). Also, LoVo and RKO cells exposed to 30 µM of methyl sartortuoate for 24, 48 and 72 h formed much fewer and smaller colonies than the 0 h group (Figure 1E,F), indicating that methyl sartortuoate inhibits growth of the two colon cancer cell lines.

### 2.2. Methyl Sartortuoate-Induced Apoptosis of Colon Cancer Cells

**Figure 2:**
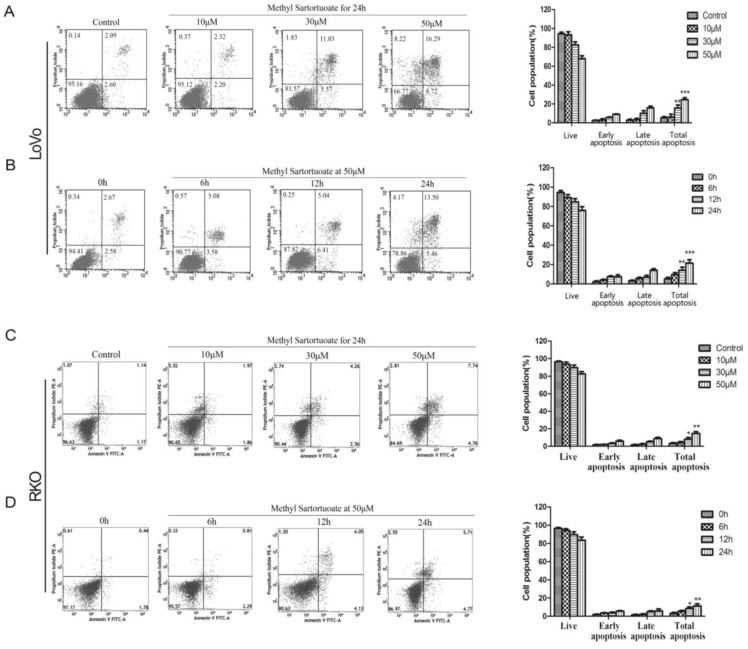
The apoptotic effects of Methyl Sartortuoate in LoVo and RKO cells. (**A**,**C**) Dose-and-effect results of Methyl Sartortuoate treated LoVo and RKO cells. LoVo and RKO cells were treated with Methyl Sartortuoate at the indicated concentration for 24 h. Apoptosis was examined by the annexin V flow cytometric assay method (*n* = 3). The percentage of apoptotic cells was scored after cell exposure to Methyl Sartortuoate. * *p* < 0.05, ** *p* < 0.01, *** *p* < 0.001 *versus* control group; (**B**,**D**) Time-and-effect results of Methyl Sartortuoate-treated LoVo and RKO cells by the annexin V flow cytometric assay method (*n* = 3). LoVo and RKO cells were treated with Methyl Sartortuoate at 50 µM for 0, 6, 12 and 24 h. The percentage of apoptotic cells were scored after cell exposure to Methyl Sartortuoate for the indicated time at 50 µM. * *p* < 0.05, ** *p* < 0.01, *** *p* < 0.001 *versus* 0 h.

The effect of methyl sartortuoate on the induction of apoptosis in LoVo and RKO cells was examined using Annexin V/PI staining. Following exposure to methyl sartortuoate at concentrations of 10, 30 and 50 µM, the apoptotic population was significantly higher than control cells (*p* < 0.01; Figure 2A,C). When LoVo and RKO cells were treated for 6, 12 and 24 h with 50 µM methyl sartortuoate, the apoptotic cell populations also were significantly higher than control cells (*p* < 0.01; Figure 2B,D).

**Figure 3:**
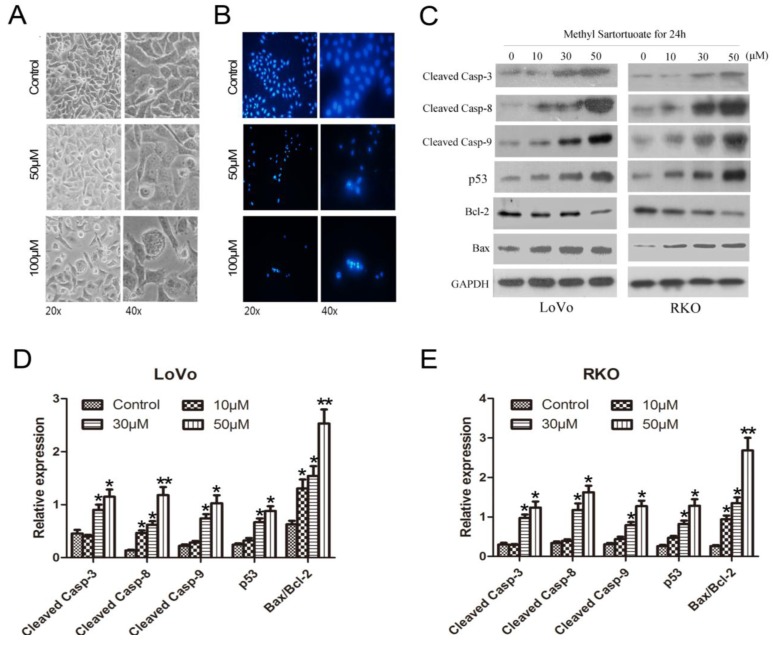
Methyl sartortuoate induces colon cancer cell apoptosis by activated apoptosis-related proteins expression. (**A**) Morphological changes in the methyl sartortuoate-treated LoVo cells were examined after treatment with 50 and 100 µM methyl sartortuoate for 24 h; (**B**) Pretreated cells were stained with DAPI for 5 min and cell shrinkage and pyknosis was visible under a fluorescence microscopy; (**C**–**E**) Cells were treated with or without various concentrations of Methyl Sartortuoate for 24 h. Cleaved Caspase-3, -8, p53, Bcl-2 and Bax levels were determined by western blotting. The relative expression of band for Cleaved Caspase-3, -8, p53, Bcl-2 and Bax proteins is indicated by Western blots, and the bar is the standard deviation (*n* = 3). * *p* < 0.05 and ** *p* < 0.01 *versus* control group.

Morphological changes in the methyl sartortuoate-treated LoVo cells were examined after treatment with 50 and 100 µM methyl sartortuoate for 24 h. In comparison with vehicle, LoVo cell numbers decreased and the cells became smaller, round, and floated. These changes were concentration-dependent (Figure 3A). DAPI staining showed that LoVo cells incubated continuously with methyl sartortuoate for 24 h started to change in shape, becoming shrunken and rounded. As shown in Figure 3B, control cells exhibited intact nuclei, while cells treated with methyl sartortuoate showed significant nuclear fragmentation.

Caspase activation was assessed using Western blotting analysis. Exposure of LoVo and RKO cells to methyl sartortuoate at 10, 30, 50 µM for 24 h resulted in progressively increased levels of cleaved caspase-3, cleaved caspase-8 and cleaved caspase-9 expression (Figure 3C–E, *p <* 0.05), suggesting the involvement of these caspases in the induction of apoptosis. As shown in Figure 3C–E, exposure of LoVo and RKO cells also resulted in decreased expression of Bcl-2 and up-regulated Bax and p53, increased the ratio of Bax/Bcl-2 and also induced apoptosis (*n* = 3, *p <* 0.01).

### 2.3. Methyl Sartortuoate Induces G2-M Phase Arrest

Cell cycle distribution was analyzed by using propidium iodide (PI) staining and flow cytometric analysis. Following exposure to methyl sartortuoate, a significant population of cells was arrested in the G2-M phase. This effect was both concentration and time dependent.

**Figure 4:**
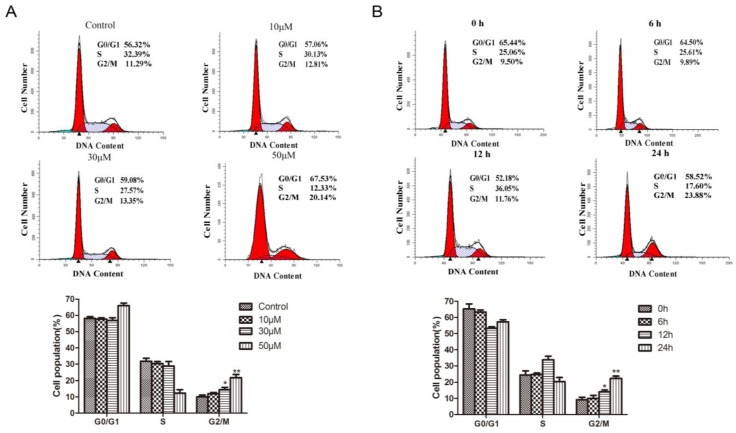
Methyl Sartortuoate causes cell cycle G2/M arrest in human colon cancer LoVo cells. LoVo cells were plated overnight and subsequently treated with Methyl Sartortuoate. At the end of treatment durations, cells were collected and either were stained with saponin/propidium iodide and analyzed for cell cycle distribution by flow cytometry. (**A**) At concentrations ranging from 0 to 50 µM in DMSO for 24 h. * *p* < 0.05, ** *p* < 0.01 compared with control group; (**B**) At a concentration of 50 µM Methyl Sartortuoate for the indicated time from 0 to 24 h, the data from three independent experiments were presented as mean ± SD; * *p* < 0.05, ** *p* < 0.01 *versus* 0 h.

As shown in Figure 4A, the population of G2-M phase LoVo cells following exposure to methyl sartortuoate-treatment for 24 h at 10, 30 and 50 µM was (12.81 ± 1.5)%, (13.35 ± 0.9)% and (20.14 ± 1.8)%, respectively compared with (11.29 ± 0.78)% in the vehicle control (*p* < 0.01). Indeed, methyl sartortuoate-treatment led to a dose-dependent induction of cell cycle arrest in the G2/M phase arrest. Exposure of LoVo cells to 50 µM methyl sartortuoate for 6, 12 and 24 h resulted in G2-M phase populations of (10.02 ± 2.1)%, (11.96 ± 0.8)% and (23.88 ± 2.48)%, respectively, compared with (9.50 ± 1.0)% with vehicle control (*p* < 0.01; Figure 4B).

**Figure 5:**
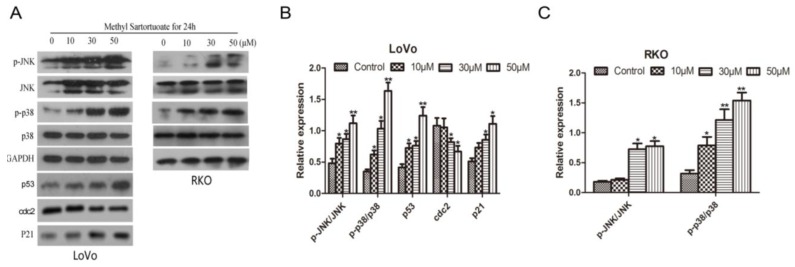

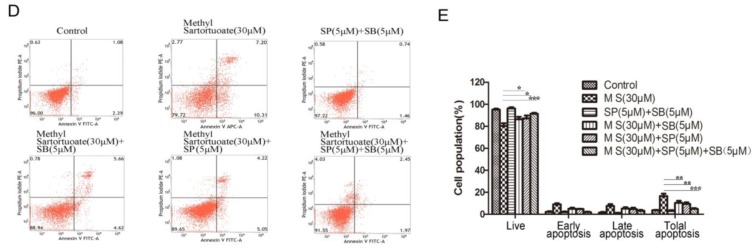
Methyl Sartortuoate induced LoVo cell apoptosis by activated JNK and P38 pathway. (**A**–**C**) Methyl Sartortuoate activated p-p38 and p-JNK in LoVo and RKO cells, and p53, p21, cdc2 proteins in the LoVo cells were measured. The relative expression of bands for p-p38, p-JNK, p53, p21 and cdc2 proteins indicated by Western blots, and the bar is the standard deviation (*n* = 3). * *p* < 0.05 and ** *p* < 0.01 *versus* control group; (**D**,**E**) LoVo cells were pre-incubated for 1 h in the presence or absence of SP600125 (5 µM), SB203580 (5 µM), and 30 µmM Methyl Sartortuoate was added for an additional 24 h. Apoptosis was examined by the annexin V flow cytometric assay method. The percentage of apoptotic cells was scored after cell exposure to Methyl Sartortuoate and was shown as the mean ± SD from three independent experiments. * *p* < 0.05, ** *p* < 0.01 and *** *p* < 0.001 compared with indicated group.

The expression levels of proteins that regulate the G2/M phase transition were measured by western blotting analysis. As shown in Figure 5A,B, cdc2 plays important roles in G2/M cycle progression, and was markedly down-regulated in cells treated with methyl sartortuoate at 10, 30, and 50 µM for 24 h, and the expression level of p21, one cdc2 inhibitor, increased prominently (*n* = 3, *p <* 0.05). Also the expression of p53, as a transcription factor that up-regulates a number of important cell cycle-modulating genes such as p21 Waf1/Cip1, was up-regulated.

### 2.4. Methyl Sartortuoate Induces JNK and p38 Downstream Signaling Pathways

**Figure 6:**
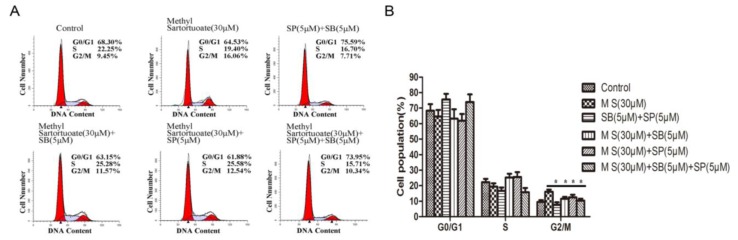

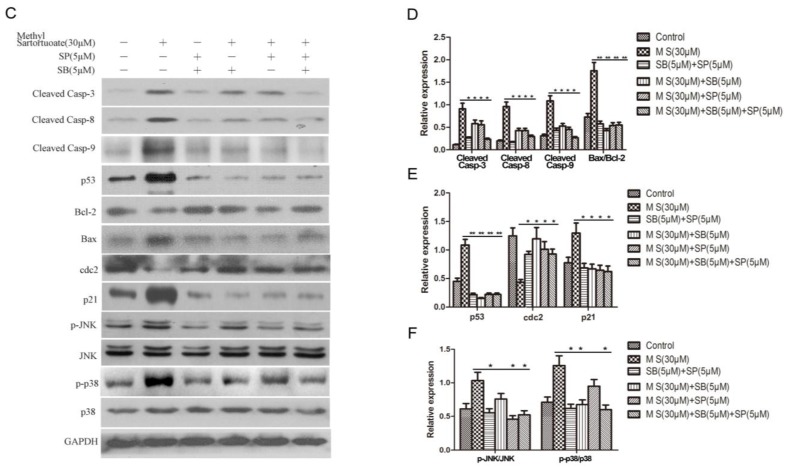
Methyl Sartortuoate induced LoVo cells apoptosis by activated JNK and P38 pathway. (**A**,**B**) Cell cycle distribution was examined by flow cytometric assay method (*n* = 3). * *p* < 0.05 *versus* indicated group; (**C**–**F**) LoVo cells were pre-incubated for 1 h in the presence or absence of SP600125 (5 mM), SB203580 (5 mM), and then treated with 30 µmM Methyl Sartortuoate for 24 h, followed by western blotting analysis of apoptosis-related proteins and cell cycle-related proteins. SP, SP600125, SB, SB203580, M S, Methyl Sartortuoate. The relative expression of band for apoptosis-related proteins, cell cycle-related proteins and p-p38, p-JNK proteins indicated by Western blots, and the bar is the standard deviation (*n* = 3). * *p* < 0.05 and ** *p* < 0.01 *versus* control group.

Western blotting analysis showed evidence that exposure to methyl sartortuoate 10, 30, and 50 µM for 24 h, might up-regulate phospho-JNK and phospho-p38 expression and abolish JNK or p38 basal activation (Figure 5A–C, *p <* 0.01). To further determine whether JNK and p38 downstream signaling pathways are involved in the methyl sartortuoate-induced colon cancer cell apoptosis, the JNK inhibitor SP600125 and the p38 MAPK inhibitor SB203580 were introduced. As Figure 5D demonstrates, the JNK inhibitor SP600125 and the p38 MAPK inhibitor SB203580 significantly prevents methyl-sartortuoate-triggered apoptosis. In Figure 6A,B, also, after treatment with JNK inhibitor SP600125 and the p38 MAPK inhibitor SB203580, it reverses the cell cycle arrest induced by methyl sartortuoate. Western blotting data showed that co-treatment of LoVo cells with methyl sartortuoate in the presence of SP600125 and SB203580 effectively blocked methyl sartortuoate-treatment cleaved caspase-3, -8, -9, Bax, and p53 up-regulation and Bcl-2 down-regulation (Figure 6C–F). Also, western blotting analysis showed that pre-treatment with SP600125 or SB203580 remarkably reduced the expression of p21 and blocked cdc2 down-regulation. These results demonstrate that the apoptosis and G2-M phase arrest of LoVo cells induced by methyl sartortuoate is mediated by the activated JNK and p38 pathway (*n* = 3, *p <* 0.05).

### 2.5. Methyl Sartortuoate Inhibits Growth of Colon Cancer in Vivo

**Figure 7:**
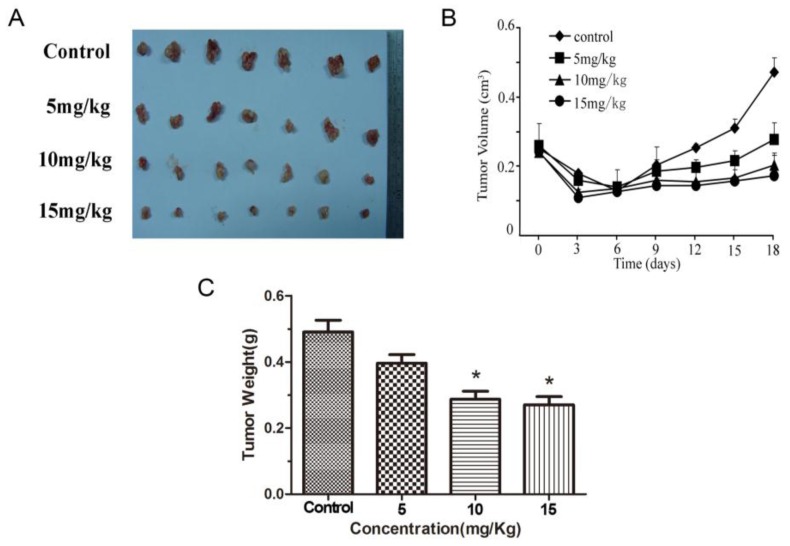

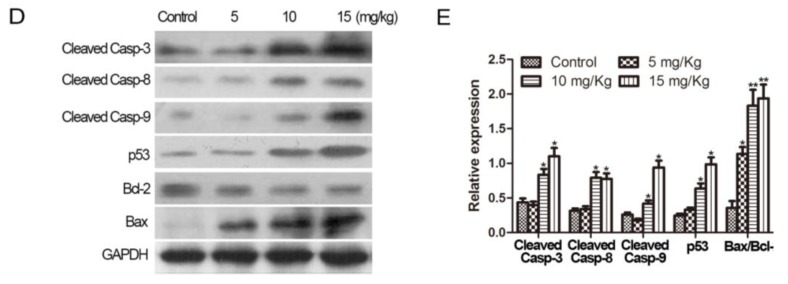
Methyl Sartortuoate at the indicated concentrations inhibited the growth of LoVo cells in nude mice. (**A**) LoVo cells were subcutaneously inoculated into the right flank of nude mice. The mice were randomly divided into four groups (*n* = 7) and treated intraperitoneally with Methyl Sartortuoate (5, 10, 15 mg/kg) or DMSO (dissolved in sodium chloride, control) every three days for 18 days. The resulting tumors were excised from the animals after treatment; (**B**) Tumor volume was measured during the experiment in the indicated days; (**C**) Tumor weight was measured after mice being sacrificed. The data experiments were presented as mean ± SD. * *p* < 0.05 compared with DMSO-treated group; (**D**,**E**) The apoptotic proteins expression in tumor tissue of mice were measured by Western blots. The relative expression of band for apoptosis-related proteins indicated by Western blots, and the bar is the standard deviation (*n* = 3). * *p* < 0.05 and ** *p* < 0.01 *versus* control group.

Tumor volumes and weight were both significantly lower in animals exposed to methyl sartortuoate than in DMSO-treated animals. Tumor volumes on Day 18 were 0.28 ± 0.02, 0.19 ± 0.02 and 0.18 ± 0.03 cm^3^ respectively in the 5, 10, and 15 mg groups compared with 0.49 ± 0.02 cm^3^ in the vehicle group of (*p* < 0.05; Figure 7A,B). Correspondingly, tumor weights were 0.37 ± 0.08, 0.25 ± 0.09 and 0.24 ± 0.04 g, respectively in the methyl sartortuoate-treated groups compared with 0.47 ± 0.06 g in the control group (*p* < 0.05; Figure 7C). Moreover, the apoptosis-relative proteins in tumor tissue were measured by Western blots. Treatment with methyl sartortuoate at 5, 10, and 15 mg/kg resulted in progressively increased levels of cleaved caspase-3, cleaved caspase-8, caspase-9, Bax and p53 expression, and resulted in decreased expression of Bcl-2 than those in the control group (Figure 7D,E, *n* = 3, *p <* 0.05).

## 3. Discussion

Terpenoids are found in herbs and marine natural products, and have been investigated as potential anticancer agents that have fewer side effects than traditional cytotoxic agents [6,16]. Various studies have shown this group of compounds to be effective as anticancer agents *in vivo*, and some members of the class are undergoing clinical trials [17,18]. Initial evidence suggests that terpenoids may be effective in the treatment and prevention of gastrointestinal cancers.

Methyl sartortuoate is a marine terpenoid, isolated from the soft coral of Sanya Bay [7]. In the present study, we have shown that methyl sartortuoate inhibits the proliferation of colon cancer LoVo and RKO cells and has effects on the cell cycle and cell apoptosis.

Apoptosis is thought to provide an important therapeutic target for new anticancer therapies that may act either by inducing cancer cell death or by sensitizing them to established cytotoxic agents [19]. Our study showed that exposure to methyl sartortuoate at 50 µM for 24 h cells caused LoVo cells to change in appearance. There was cell shrinkage, rounding and floating. DAPI staining showed evidence of nuclear pyknosis and karyorrhexis in methyl sartortuoate-treated tumors. We showed that methyl sartortuoate resulted in dose- and time-dependent apoptosis in LoVo and RKO cells.

It is widely reported that caspases may play a critical role in the apoptotic pathway and are widely expressed as inactive proenzymes in most cell types [20,21]. Caspase-dependent apoptosis occurs through either an extrinsic pathway leading to activation of caspase-8 or via an intrinsic pathway leading to activation of caspase-9. Both pathways share a common downstream pathway involving the activation of effector caspases and finally lead to apoptosis [22]. The Bcl-2 family proteins are key regulators of apoptosis. These include the anti-apoptotic proteins (Bcl-2, Bcl-XL, and Mcl-1) and pro-apoptotic proteins (Bax, Bad, and Bid) [22], and the expression levels of both Bcl-2 and Bax are regulated by the p53 tumor suppressor gene [23]. Our results showed that methyl sartortuoate markedly enhanced the up-regulation of cleaved caspase-8, cleaved caspase-9 and cleaved caspase-3. Activated caspase-3 in turn induced apoptosis with a decrease in Bcl-2 level. Moreover, the expression of p53 up-regulation can regulate the expression of both Bcl-2 and Bax. Our research results demonstrate that methyl sartortuoate can enhance the expression of p53, and Bax, and down-regulate the expression of Bcl-2. These findings suggest that methyl sartortuoate may be able to induce caspase-dependent apoptosis via an extrinsic pathway and an intrinsic pathway that may form a crosstalk through caspase-8-mediated cleavage of Bid to stimulate the intrinsic pathway, and that this effect may be accompanied by up-regulation of p53, which regulates the expression of both Bcl-2 and Bax.

Based on these findings, we attempted to investigate the signaling pathways involved in methyl sartortuoate-induced apoptosis. The MAPK pathway has emerged as one of the essential signaling mechanisms in cell growth inhibition and its downstream effectors are responsible for propagating the signals to promote apoptosis [24]. Activation of this pathway is thought to be involved in caspase-mediated apoptosis [25,26,27].

Western blot analysis was used for evaluating the expression of phosphorylation levels of JNK and p38 protein. We showed that, methyl sartortuoate significantly up-regulated the expression of phospho-JNK and p38 protein, and methyl sartortuoate-mediated apoptosis can be confirmed by the use of JNK inhibitor SP600125 and the p38 MAPK inhibitor SB203580, suggesting that interruption of MAPK signal network by methyl sartortuoate contributes to the growth-inhibition and survival of LoVo and RKO cells.

In eukaryotes, mitosis is dependent on the completion of DNA synthesis [28]. Cells can manage both endogenous and exogenous DNA damage though highly conservative DNA-repair and cell-cycle checkpoint signal pathways [29]. Several therapeutic agents can disrupt cell cycle regulation and impair checkpoint controls ultimately inducing growth arrest and apoptosis in cancer cells [30]. Our study showed for the first-time that methyl sartortuoate was able to induce G2-M phase arrest in LoVo cells. Base on ample evidence, the cyclin/CDK complexes are modulated by CKIs, such as p21WAF1/Cip1 (referred to as p21 hereafter), which bind to specific CDK complexes, that can largely regulate the eukaryotic cell division cycle and lead to cell cycle arrest [11]. P53 is taken as a transcription factor that up-regulates a series of important cell cycle-modulating genes such as p21 Waf1/Cip1. As a cyclin-dependent kinase inhibitor, p21 Waf1/Cip1, under the regulation of p53, can bind to the cyclin/CDK complexes inducing cell cycle arrest. In the present study, we observed that the expression of p53, p21 Waf1/Cip1 was remarkably increased in LoVo cells treated with methyl sartortuoate, which probably contributes to induce G2-M phase arrest in colorectal cancer cells. Moreover, this was confirmed by the use of JNK SP600125 and the p38 MAPK inhibitor SB203580. These results indicate that up-regulation of p53 and p21 expression, and suppression of cdc2 by methyl sartortuoate may result in G2-M phase arrest in colorectal cancer cells.

## 4. Materials and Methods

### 4.1. Materials and Cell Culture

Rabbit polyclonal antibody specific for caspase-3, Bax and Bcl-2 apoptosis regulating proteins were purchased from Abcam (Cambridge, MA, USA). Anti-caspase-8, anti-caspase-9, anti-phospho-p38 MAPK (Thr180/Tyr182), anti-p38 MAPK, anti-Phospho-JNK (Thr183/Tyr185), anti-JNK, anti-p53, anti-p21 Waf1/Cip1, anti-cdc2 and anti-glyceraldehyde-3-phosphate dehydrogenase (GAPDH) antibody were purchased from Cell Signaling Technology (Beverly, MA, USA). DMSO was purchased from Sigma-Aldrich (St. Louis, MO, USA). 3-(4,5-Dimethylthiazol-2-yl)-2,5-diphe-nyl tetrazolium bromide (MTT), propidium iodide (PI), JNK inhibitor SP600125 and p38 MAPK inhibitor SB203580 were obtained from Sigma (St. Louis, MO, USA). MTT was dissolved in phosphate-buffered saline solution (PBS) at a stock concentration of 5 mg/mL and filtered to obtain a sterilized solution. PI was dissolved in PBS at 100 mg/mL.

**Figure 8:**
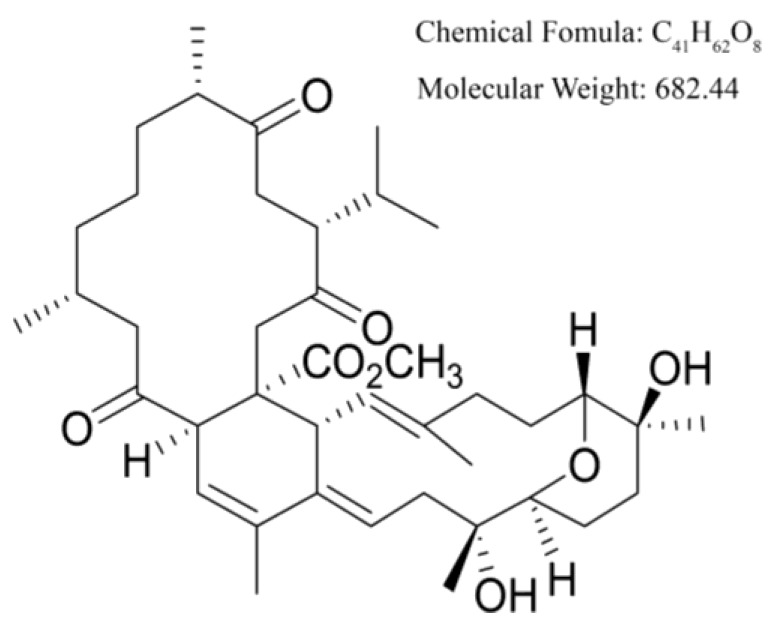
Chemical structure of Methyl Sartortuoate.

Methyl sartortuoate was provided by the School of Pharmaceutical Sciences, Sun Yat-sen University (Guangzhou, China). The chemical structure is shown in Figure 8. The compound was dissolved in DMSO at a concentration of 50 mM to generate a stock solution. The compound was further diluted in culture medium for the culture experiments.

The human colon cancer cell line LoVo and RKO (purchased from the American type culture collection (ATCC)) was maintained in RPMI-1640 supplemented with 10% fetal bovine serum (Gibco, Carlsbad, CA, USA), 100 IU/mL penicillin, and 100 µg/mL streptomycin. The culture flasks were incubated at 37 °C in a 5% CO_2_ humidified atmosphere. The media were changed every second or third day.

### 4.2. Cell Proliferation Assays

The cytotoxicity of methyl sartortuoate to LoVo and RKO cells was measured by MTT assay. Briefly, 200 µL of LoVo cells (2000 cells per well) was seeded into 96-well flat-bottomed plates and exposed to methyl sartortuoate for 24 h. This was followed by the addition of, 20 µL of 5 mg/mL MTT to each well. After incubation for 4 h, the supernatant was decanted, and DMSO was added to all wells and mixed thoroughly to ensure that all crystals were dissolved. The optical densities (ODs) of the plates were read on a ELISA reader (Molecular Device Co., Sunnyvale, CA, USA), using a test wavelength of 492 nm.

### 4.3. Colony Formation Assay

LoVo and RKO cells were plated as 10^3^ cells/well in six-well plates and maintained with or without methyl sartortuoate for 1 week. After growth, colonies were fixed with methanol for 30 min and stained with 0.1% crystal violet for visualization and counting.

### 4.4. Protein Extraction and Western Blots

LoVo and RKO cells cultures were washed with PBS and lysed with RIPA buffer containing 50 mM Tris-HCl (pH 7.4), 150 mM NaCl, 0.5 mM phenylmethanesulfonyl fluoride (PMSF), 1% Triton X-100, 1% sodium deoxycholate and 0.1% SDS. The protein concentration was determined by Bradford assay. Equal amounts of protein (30 µg/lane) were boiled at 100 °C with loading buffer for 5 min and separated by electrophoresis on 12% SDS-PAGE gels. The proteins were transferred to nitrocellulose polyvinylidene difluoride (PVDF) membranes (Bio-Rad, Indianapolis, IN, USA) and blocked in TBS buffer with 5% non-fat milk.

The blots were further incubated overnight at 4 °C with a series of primary antibodies. After washing, the membranes were incubated with HRP-conjugated secondary antibody (1:5000) for 1 h at room temperature. Labeled proteins were visualized by enhanced chemiluminescence reagents (Amersham Biosciences Piscataway, NJ, USA) and developed on X-ray film. The protein expression of phospho-JNK or phospho-P38 was normalized to the total JNK or P38, respectively. The band signals of other interesting proteins were normalized to those of GAPDH from the same samples. The relative band intensities of blots were measured using the Quantity One software (Bio-Rad).

### 4.5. Flow Cytometry Analysis for Apoptosis

Following exposure to methyl sartortuoate apoptosis was quantified using Vybrant Apoptosis Assay Kit 2 (Molecular Probes, Eugene, OR, USA) as per the vendor’s protocol. Briefly, at the end of the exposure to methyl sartortuoate non-adherent and adherent cells were collected after brief trypsinization. The cells were washed once with ice-cold PBS, and subsequently stained with Annexin V and PI. Stained cells were analyzed by flow cytometry using fluorescence-activated cell sorting analysis core facility of Medicine Research Center of Sun Yat Sun Memorial Hospital.

### 4.6. Morphological Detection of Apoptosis

Morphological evaluation of apoptotic cell death was performed using 4ʹ-6-Diamidino-2-phenylindole (DAPI) according to the manufacturer’s instructions (Beyotime Institute of Biotechnology). Briefly, 5 × 10^4^ LoVo cells were seeded onto coverslips in six-well culture plates. After treatment with 50 µM of methyl sartortuoate for 24 h, the cells were fixed at 4 °C overnight. The following day, the cells were stained with 500 µL of DAPI for 5 min and then subjected to fluorescence microscopy.

### 4.7. Flow Cytometry Analysis for Cell Cycle Distribution

Following exposure to methyl sartortuoate the cells were harvested by brief trypsinization, washed twice with ice-cold PBS, and the resulting cell pellets were collected. Approximately 0.5 × 10^5^ cells in 0.5 mL saponin/PI solution were incubated at 4 °C for 24 h in the dark. Cell cycle distribution was then analyzed by flow cytometry (Becton-Dickson) through 10,000 events and the ModFit LT 2.0TM software (Verity Software House Inc., Topsham, ME, USA) was used to assess the cell cycle distribution patterns (G0/G1, S and G2-M phases).

### 4.8. Mice Tumor Model

Athymic nude mice (BALB/c nu/nu, 6-week old females) were purchased from the Vital River Laboratories (China). The protocol was approved by the Animal Ethical and Welfare Committee of Sun Yat-sen University. All surgeries were performed under sodium pentobarbital anesthesia, and all efforts were made to minimize suffering.

Tumor xenografts were established by injecting 1 × 10^6^ LoVo cells into the subcutaneous tissue in both flanks of nude mice. When the tumor sizes reached ~0.25 cm^3^, mice were randomly divided into four groups of seven animals and treated intraperitoneally with methyl sartortuoate (5,10 and 15 mg/kg), DMSO (negative control) dissolved in NaCl every 3 days for18 days.

The mice were kept in pathogen-free environments and checked and recorded every 3 days. Tumor volume was, determined by the formula: 0.5 × length × width^2^. All animals were sacrificed on day 18. Tumors were removed and weights were measured.

### 4.9. Statistical Analysis

Statistical analyses were performed using SPSS version 13.0 (SPSS Inc., Chicago, IL, USA). Data are presented as the mean ± standard deviation (SD). Differences between control and treated groups was determined using Student’s *t* tests or one-way analysis of variance (ANOVA) followed by Bonferroni *t* tests. Values of *p* < 0.05 were considered statistically significant.

## 5. Conclusions

In conclusion, we report here for the first time evidence for the anti-colon cancer activity of methyl sartortuoate *in vitro* and *in vivo*. Methyl sartortuoate was found to inhibit growth of LoVo and RKO cells and induce apoptotic death, in a concentration-and time-dependent manner, with an IC_50_ value of 48 and 51 µM. Methyl sartortuoate also showed antitumor activity in the NOD-SCID nude mice inoculated with LoVo. We propose that methyl sartortuoate inhibits colon cancer cell growth and induces apoptosis through the activation of caspase-8, caspase-9 and caspase-3, accompanied by the inactivation of Bcl-2 and up-regulation of p53 and Bax, this results in cell cycle arrest in LoVo cells at the G2-M phase. The mechanisms of action of methyl sartortuoate appear to involve phosphorylation of JNK and p38 signalling. Further studies are needed to clarify the pathogenetic mechanisms of colon cancer and support the further development of methyl sartortuoate for use in colon cancer.
